# A review of malaria vaccine clinical projects based on the WHO rainbow table

**DOI:** 10.1186/1475-2875-11-11

**Published:** 2012-01-09

**Authors:** Lauren Schwartz, Graham V Brown, Blaise Genton, Vasee S Moorthy

**Affiliations:** 1 Initiative for Vaccine Research, Department of Immunization, Vaccines & Biologicals, World Health Organization, Avenue Appia 20, 1211-CH 27, Geneva, Switzerland; 2 Johns Hopkins Bloomberg School of Public Health, Johns Hopkins University, Baltimore, Maryland, USA; 3 Nossal Institute for Global Health, University of Melbourne, Carlton, Victoria 3010, Australia; 4 Infectious Disease Service & Department of Ambulatory Care and Community Medicine, University Hospital, Lausanne, Switzerland; 5 Swiss Tropical and Public Health Institute, University of Basel, Socinstrasse 57, Postfach, 4002 Basel, Switzerland; 6 Nuffield Department of Clinical Medicine, University of Oxford, John Radcliffe Hospital, OX3 9DU, Oxford, UK

## Abstract

Development and Phase 3 testing of the most advanced malaria vaccine, RTS,S/AS01, indicates that malaria vaccine R&D is moving into a new phase. Field trials of several research malaria vaccines have also confirmed that it is possible to impact the host-parasite relationship through vaccine-induced immune responses to multiple antigenic targets using different platforms. Other approaches have been appropriately tested but turned out to be disappointing after clinical evaluation.

As the malaria community considers the potential role of a first-generation malaria vaccine in malaria control efforts, it is an apposite time to carefully document terminated and ongoing malaria vaccine research projects so that lessons learned can be applied to increase the chances of success for second-generation malaria vaccines over the next 10 years.

The most comprehensive resource of malaria vaccine projects is a spreadsheet compiled by WHO thanks to the input from funding agencies, sponsors and investigators worldwide. This spreadsheet, available from WHO's website, is known as "the rainbow table". By summarizing the published and some unpublished information available for each project on the rainbow table, the most comprehensive review of malaria vaccine projects to be published in the last several years is provided below.

## Background

Few recent malaria vaccine review articles have attempted a comprehensive outline of all clinical trials that have occurred globally. The field has grown to such an extent that it is now very difficult to summarize all projects in a single review. The increase in funding over the last 10 years has allowed over 40 vaccine projects to reach the clinical trial stage. This manuscript is a comprehensive review of malaria vaccine clinical projects written in recent years, though even here it is possible that not every project has been included.

WHO compiles the "rainbow table" spreadsheet, a comprehensive publicly available collation of global malaria vaccine project activity with input from funders, sponsors and investigators [1]. For this review, published papers related to each project from the rainbow table were obtained, and clinical trial registry information and conference abstracts were read, where papers are not yet published.

As this review is based on projects which have reached the clinic, it is best seen as a documentation and discussion of projects which have reached that stage. This review does not present the status of current pre-clinical malaria vaccine development, other than some discussion on links between pre-clinical and clinical results for the projects outlined below.

### Status of malaria control

Between 2000 and 2009 there have been major gains in malaria control in many malaria-endemic countries, including many in Africa [2]. Total estimated numbers of deaths related to malaria have dropped from about 1 million in 2000 to about 780,000 in 2009. The numbers of clinical cases are estimated at 225 million globally by WHO [3]. These gains are associated with scaling-up of existing WHO recommended malaria control measures, including long-lasting insecticidal nets (LLIN), indoor residual spraying programmes (IRS) and access to artemisinin combination therapy(ACT)[3]. There has also been a shift towards use of rapid diagnostic tests and away from presumptive treatment of malaria. Success of malaria control is vulnerable to the emergence of resistance to artemisinins and insecticides and depends on the vital imperative for sustained malaria control funding. Of the five species of Plasmodium that are known to cause disease in humans, two have received attention for vaccine development. Over 90% of malaria-related deaths are caused by *Plasmodium falciparum*, and there is a similar dominance for *P. falciparum *projects in the malaria vaccine landscape. A single *Plasmodium vivax *project is currently in the clinic; this is listed at the end of the review.

### Rationale and goals for malaria vaccine development

Many lines of evidence indicate that humans can be vaccinated against malaria. Individuals born in endemic areas who survive the first years of exposure continue to develop parasitaemia on natural exposure, but become resistant first to severe, life-threatening malaria and then to clinical disease. Frequent re-exposure is required to maintain this condition of immunity with infection (concomitant immunity). Transfer of gamma-globulin fractions from semi-immune to naïve humans mitigates malaria disease [4,5], demonstrating that clinical protection from malaria is possible, and that immunoglobulin targeting malaria antigens can play a critical role. Inoculation of humans with irradiated sporozoites by mosquito bite can prevent the emergence of blood-stage infection after subsequent experimental challenge [6,7], demonstrating the possibility of inducing high level protection against infection under experimental conditions. In endemic areas with natural exposure, sterile immunity rarely if ever develops. Perhaps most importantly and significantly, the candidate vaccine RTS,S/AS can induce clinical efficacy in the 25-60% range in different malaria endemic settings. Thus, the question of feasibility of malaria vaccination has progressed to an assessment of the public health role of RTS,S vaccination and the possibility of developing even more efficacious second-generation vaccines.

The Malaria Vaccine Technology Roadmap launched in 2006 expressed intermediate and aspirational community goals for vaccine benefits. First, a vaccine with 50% efficacy against severe disease and malaria-related mortality protecting for more than 12 months, and secondly a longer-lasting vaccine with 80% efficacy against clinical malaria [8]. The primary refinement introduced by the malaria eradication R&D agenda setting process during 2009-2010 is the confirmation that for elimination and global eradication, impact on transmission rather than morbidity is the paramount efficacy outcome. There is general agreement that malaria eradication is not possible with the currently available tools. Development of a highly efficacious malaria vaccine which dramatically reduces transmission would be a transformative tool that could enable future eradication.

### Overview of current status of malaria vaccine clinical development

Funding has increased substantially over the last 10 years with contributions from agencies such as the Bill and Melinda Gates Foundation (particularly through PATH Malaria Vaccine Initiative), the US National Institute for Allergy and Infectious Disease, European Union DG RTD, United States Agency for International Development, Wellcome Trust, Medical Research Council UK, the European Vaccine Initiative (formerly EMVI), European and Developing Countries Clinical Trials Partnership and WHO [9]. There was a lag of several years before the funding led to a large increase in the numbers of clinical trials.

Pre-erythrocytic malaria vaccines designed to produce sterile protection can be terminated at the challenge trial stage if no efficacy is demonstrated, whereas blood stage vaccines generally progress to field evaluation for proof of concept of clinical effect; thus the timelines to reach proof of concept for blood-stage vaccines have been much longer than for pre-erythrocytic vaccines. This may explain why there are more potential blood-stage vaccines than other life-cycle stages in clinical evaluation, while many pre-erythrocytic concepts have been tested to failure and terminated, and others are in the pipeline. Substantial investment in field trial sites for blood-stage (and pre-erythrocytic) vaccine evaluation took many years, but is now bearing fruit. Because of the high cost of large-scale clinical trials, major efforts continue to find surrogate predictors of efficacy, such as reduction in incidence of infection, that could be used to prioritize candidates for the long and expensive clinical field trials. Others are reassessing the evaluation paradigm for blood-stage vaccines and considering use of the challenge trial model, together with functional assays as markers for the down-selection step. Sexual stage and mosquito antigen vaccines are receiving renewed attention but are still grossly under-represented in clinical project portfolios.

*Plasmodium falciparum *is a highly immuno-evasive, multi-stage protozoal parasite with several antigenically distinct mosquito vector and human stages. Molecular understanding of naturally acquired immunity remains in its early stages and there are few well characterized molecular targets that could be selected with confidence by vaccine developers as being the basis for subunit-based protection. Thus it is no surprise that many of the necessarily empiric projects outlined below have not yielded clinical efficacy [10]. Despite the success of the *P. falciparum*, *A. gambiae *and human genome projects, there has been little translation of antigenic targets from post-genomic antigen discovery to clinical evaluation, partly because of the problems of selecting appropriate targets, and the lack of robust and reliable predictive animal models. What is perhaps more surprising, given the daunting nature of the task, is that malaria vaccine developers have produced startling progress in several areas.

The first human anti-parasite vaccine will be considered for licensure by regulators in the next few years, as RTS,S/AS01E progresses through clinical evaluation in a pivotal Phase 3 trial. This product was developed through a partnership between GSK and PATH Malaria Vaccine Initiative, with funds from the Gates Foundation to MVI. Multiple other projects have yielded a degree of efficacy, notably prime-boost pre-erythrocytic projects, and very significant expertise and infrastructure has been developed in African field trial sites. The recent reductions in malaria transmission in Africa, if sustained, will render field efficacy trials more difficult in many current research settings. For some funders, this drop in transmission together with the partial success of RTS,S has shifted emphasis away from blood-stage vaccines towards pre-erythrocytic and sexual stage/mosquito (SSM) antigen vaccine development, emphasising the role they could play in further reduction of transmission. Clinical evaluation of SSM vaccines will however be challenging because these vaccines confer efficacy to humans only at the population level and thus traditional individually randomized trial designs will not apply without major modification [11,12]. An additional problem is the lack of knowledge of the relationship between the effect of a vaccine at an individual level, such as rendering an individual 80% less infectious to mosquitoes, and the effect on transmission. For example, the effect on transmission of a vaccine that makes x% of people y% less infectious is not known, although some insights can be gained from modelling.

Success with the novel adjuvants used with RTS,S is now coupled with an interest in various conjugation or particulate technologies in attempts to overcome the poor immunogenicity of soluble monomeric recombinant malaria proteins [13]. Identification of scalable manufacturing processes remains challenging and laborious for many *P. falciparum *antigens that have complex multiply disulphide bonded structures and conformationally dependent induction of protective IgG. The success of malaria control also raises the bar for the expected performance of a malaria vaccine. Finally an increased emphasis on *P. vivax *R&D has been announced by some funders, and this has already led to evaluation of one new *P. vivax *candidate in Phase 1 [14].

All but one of the projects detailed below is based on a *P. falciparum *antigen. It is likely there will be more emphasis on *P. vivax *in future as this species becomes relatively more important from the public health perspective. As many of the projects involve multiple partners and funders we have chosen not to provide details of the vaccine development partnerships here, to avoid providing incomplete information. These can easily be found through the references, the clinical trial registry sites and on the WHO malaria vaccine rainbow table [1].

The classification of projects below is by species, with *P. falciparum *projects first, and by life-cycle stage. For each life-cycle stage we report currently active projects first, followed by discontinued/inactive projects. It is important to note that many of the discontinued/inactive projects have contributed greatly to understandings of vaccine science [10], and that some will return to the clinic either in new iterations or without substantial changes if further funding is obtained. There is a separate section for combination vaccines including multiple life-cycle stages. Combination or polyvalent/multi-epitope constructs focusing on one life-cycle stage are highlighted as such within the text.

### *Plasmodium falciparum *pre-erythrocytic projects

There is no consensus that pre-erythrocytic immune responses gained through natural exposure, whether antibody or cell-mediated immunity (CMI), contribute substantially to naturally acquired immunity [15], but the irradiated sporozoite model proves that humans can be protected by immune responses to sporozoite and liver-stage parasites. In this sense, sterile immunity conferred by pre-erythrocytic subunit vaccines or attenuated whole organisms would not be mimicking naturally acquired immunity, yet this induced "non-natural" immunity could be more effective than is seen in nature, whether it supplements or replaces that usually seen in an endemic area. Candidate vaccine antigens from the pre-erythrocytic stages may be the targets of antibodies that prevent sporozoite invasion of hepatocytes or the targets of cellular immune responses that kill infected hepatocytes. A completely effective pre-erythrocytic vaccine would inactivate the parasite before it left the liver, leading to sterile immunity and prevention of disease. This goal may or may not be achievable with the vaccines that are currently being evaluated, but a partially effective vaccine could decrease the incidence of new infections, and decrease the number of merozoites exiting the liver, by decreasing the number of sporozoites entering the liver or killing parasites within hepatocytes, leading to clinical benefits analogous to the direct effects of insecticide treated bed nets. Partially effective pre-erythrocytic vaccines could lead to reductions in both the size and frequency of blood-stage inocula, which could result in reductions in mild disease, severe disease and mortality. A reduced size of inoculum that lengthens the gap between infection and patency may allow time for boosting other immune responses that contribute to clinical immunity. Reduction in multiplicity of infection would be another predicted and potentially important effect of such partially effective vaccines.

#### CS protein

The Plasmodium circumsporozoite protein (CS) is expressed during the sporozoite and early liver stages of parasitic infection [16]. This protein is involved in the adhesion of the sporozoite to the hepatocyte and invasion of the hepatocyte. Anti-CS antibodies have been shown to inhibit parasite invasion and are also associated with a reduced risk of clinical malaria [17,18] in some studies, though the relative importance of anti-CS responses in naturally acquired immunity remains controversial. Antibodies raised through immunization with only the conserved Asparagine-Alanine-Asparagine-Proline (NANP) amino acid repeat sequence, the immunodominant B-cell epitope from *P. falciparum *CS, are capable of blocking sporozoite invasion of hepatocytes [19]. The demonstrated protective role of vaccine-induced anti-CS responses and the fact that CS is the predominant surface antigen of sporozoites [19], have made CS the most popular antigen for use in pre-erythrocytic vaccine candidates. In this regard it is set apart from other candidate vaccine antigens, as the lead antigen. Evidence for antigen specific vaccine-induced efficacy against morbidity is far stronger for CS than any other antigen.

#### RTS,S/AS01E

Clinical development of RTS,S/AS01E has been reviewed extensively [20-22]. This is by far the most advanced candidate malaria vaccine, is the only one in Phase 3 evaluation, and is at least 5-10 years ahead of all other projects. RTS,S/AS01E has demonstrated 51% efficacy (95%CI 29-66) in reducing the rate of all episodes of clinical malaria over fifteen months of follow-up in a Phase 2 trial in children aged 5-17 months resident in Kilifi, Kenya [23]. Immunologic analyses indicate that high titre anti-CS IgG are most strongly associated with RTS,S-mediated protection, with an important additive component from CS-specific Th1 cells. One recent study highlighted a correlation between CS-specific TNFα(+) CD4 (+) T cells and reduced morbidity, which requires confirmation in other studies [24]. The ongoing Pivotal Phase 3 trial started in May 2009 and has enrolled 15,460 children over 6,000 of whom are in the 6-14 week EPI co-administration age group. The full trial results are expected in 2014 and will include the following information: safety and reactogenicity of a vaccine containing a novel adjuvant, co-administration data with pentavalent DTwP/HepB/Hib and OPV, efficacy in multiple transmission settings, efficacy data over 30 months of follow-up, an 18 month booster dose and efficacy against severe, life-threatening malaria. The first of 3 sets of results from the Phase 3 trial was published on 18 Oct 2011 and was in line with expectations from the Phase 2 trials [25,26]. The trial, conducted at 11 trial sites in seven countries across sub-Saharan Africa, reported that RTS,S reduced the incidence of all episodes of clinical malaria by 55% (95%CI 51-59) when evaluated over 12 months following the third dose. This analysis was performed on data from the first 6,000 children aged 5 to 17 months. A primary analysis for severe malaria efficacy was planned when 250 cases accrued in both the 5-17 month and 6-14 week age groups of the trial. This analysis reported an efficacy of 35% (95%CI 16-49) with variable follow-up from zero to 22 months after the third dose. There are many lessons to be learned from the RTS,S trials including the major contribution of sporozoite challenge trials, the importance of adjuvant, dose and schedule optimization, and the need to use particulate structures to enhance immunogenicity.

There is a standing WHO advisory group, known as the Joint Technical Expert Group, which will review data as they become available from the Phase 3 trial. Policy recommendation timings are data-driven. Depending on the full trial results expected in 2014, WHO recommendation for use may occur in 2015.

#### Adenovirus (Ad35) vectored CS

RTS,S/AS01E induced very potent anti-CS Ig responses, modest CD4+ γ-interferon T cell responses and low or absent CD8 responses. The approach considered most likely to improve upon CS-mediated protection would be to employ a prime-boost combination of RTS,S/AS01 with a CD8-inducing CS vaccine. The non-replicating adenovirus 35 vector encodes the CS protein. In preclinical development the vaccine induced strong IFN-γ responses in mice including CD8+ responses, thought to be important for protective immunity in humans [27,28]. Phase 1 human studies examining safety and immunogenicity have occurred at Stanford and Vanderbilt Universities [29] with a Phase 1b study in Burkina Faso now completed [30]. The potential interference of antibodies induced by previous natural exposure to Ad 5 is not known, but one attraction for adenovirus 35 is the much lower seroprevalence of antibodies to this virus than adenovirus 5 [31]. The HIV STEP trial results [32] are thought by some to represent a roadblock to the future of adenovirus 5 as a vector for prophylactic vaccine.

#### Ad35 vectored CS in prime-boost with RTS,S/AS01E

Crucell announced in 2009 that they have agreed to work with GSK to evaluate adenovirus 35 CS/RTS,S prime-boost combinations, hopefully reproducing the promising non-human primate results seen with this approach [33]. Regimens based on RTS,S/AS01, but including rational prime-boost additions or additional antigens in combination, are the most likely near-term possibilities for improving efficacy induced by RTS,S/AS01 alone. The Phase 1/2a sporozoite challenge study began in August 2011 comparing one dose of Ad35 CS followed by two doses of RTS,S/AS01 with 3 doses of RTS,S/AS01 alone [34].

#### Multiple epitope constructs

Some vaccine strategies use components of multiple pre-erythrocytic antigens in one vaccine to elicit a broad immune response intended to prevent blood-stage infection. This approach is intermediate between traditional single antigen constructs and combinations of whole antigens. One example is the ME-TRAP construct, a pre-erythrocytic fusion antigen consisting of 17 B cell, CD4+ and CD8+ T cell epitopes from six *P. falciparum *antigens fused to the T9/96 allele of TRAP (thrombospondin-related adhesion protein) pre-erythrocytic antigen. It includes a single *Plasmodium berghei *CD8+ T cell epitope (Pb9) for potency studies in mice. The vaccine construct is based on the attractive concept of IFN-γ mediated elimination of infected hepatocytes [35,36]. TRAP is another protein expressed both on the surface of sporozoites [37] and within infected hepatocytes [38].

#### AdCh63/MVA ME-TRAP

During preclinical development of this vaccine, a prime-boost sequence of simian adenovirus (AdCh63) encoding for ME-TRAP boosted with modified vaccinia virus Ankara (MVA) encoding the same construct elicited exceptionally strong and long-lasting CD8+ T cell responses [36,39]. Initial studies had used human adenoviruses, but previous exposure to the vector could produce neutralizing antibodies that would interfere with immunogenicity. This problem is overcome through use of simian adenovectors to which humans do not have cross-reactive antibodies. In the UK a Phase 1 study and a Phase 2 sporozoite challenge study should be completed during 2011 [40,41]. The first adult Phase1 Kenyan study had started by November 2010 and a Phase 1b field trial in the Gambia has also occurred [1]. Prime-boost approaches including adenoviruses appear particularly promising for achievement of the long desired outcome of strong induction of CD8 T cell responses in humans; the strain of adenovirus used may be vital to the immunogenicity and efficacy afforded, both in malaria-naïve individuals and in malaria-endemic settings.

#### Polyepitope DNA EP1300

This pre-erythrocytic DNA vaccine includes multiple epitopes with linker sequences from four pre-erythrocytic antigens, CS, SSP2/TRAP, Liver-stage antigen 1 (LSA-1) and Exported protein 1 (Exp-1) and is administered via electroporation. The strategy is being assessed in Phase 1a studies in naïve volunteers in the US which started during 2010 [42]. Few further details are available. The immunogenicity of DNA vaccines in animal models has generally not been reproduced in humans. Augmentation has previously been attempted with various intradermal, subcutaneous and intramuscular delivery devices [43]. Electroporation has augmented immune responses impressively in some animal models [44,45] and in a Phase 1 clinical trial of an HIV vaccine construct [46]. It remains to be seen whether tolerability will be acceptable and immunogenicity sufficient for prophylactic paediatric vaccination in humans. This project also touches on the important question of how to best ensure immunogenicity of multiple epitopes or antigens in DNA-based approaches.

### Whole organism approaches

#### PfSPZ: metabolically active, non-replicating malaria sporozoite vaccine

Using the knowledge that volunteers can be protected from sporozoite challenge by immunization via bites from > 1,000 irradiated *P. falciparum*-infected mosquitoes, a biotech company has developed an approach using injection of metabolically active, non-replicating whole *P. falciparum *sporozoites thawed from long-term storage in liquid nitrogen. The vaccine should confer sterilizing immunity in principle, but challenges arise from manufacturing and scaling up of the product, dosage and administration methods, and the logistics of delivering a vaccine that is cryopreserved in liquid nitrogen [47-49]. In initial Phase 1/2a studies to examine the safety and immunogenicity of different dosages and i.d. and s.c. routes of administration in malaria naïve volunteers [50], two out of 44 subjects challenged were completely protected, but refinements to the dose, route or schedule or improvements in adjuvants are required for a vaccine likely to have significant impact on morbidity. With such a novel vaccine technology, it is likely that several further Phase 1 trials will be necessary to identify a regimen that can replicate the results previously seen when irradiated sporozoites were delivered by mosquito bite. A follow-on Phase 1/2a of i.v. administration is underway [51].

#### Genetically attenuated sporozoites

Another whole organism approach is inoculation of genetically attenuated parasites. A multi-institutional partnership is developing and testing genetically attenuated sporozoites as whole organism pre-erythrocytic immunogens [52,53]. A Phase 1 study has occurred; no report had been made at the time of writing.

### Discontinued/inactive pre-erythrocytic projects

#### FP9 CS/MVA CS

This strategy assessed whether priming with an attenuated fowlpox strain (FP9) expressing the pre-erythrocytic CS protein and boosting with MVA also coding for the CS protein would elicit strong cellular responses. A Phase 1/2a study in malaria naïve subjects in the UK found that while the vaccine regimen was safe, the T cell response was modest and MVA did not boost this T cell response convincingly. When challenged with sporozoites, there was no protection of study subjects and no partial protection, defined by a delay in time to parasitaemia [54]. Conversely, in a Phase 1b study in Gambian adults, IFN-γ producing CD4+ and CD8+ cells were substantially elevated after boosting with MVA with the most likely explanation being priming of the response by natural infection [55]. Interestingly this result was predicted by earlier pre-clinical research on natural priming performed by a group at New York University [56].

#### DNA CS/MVA CS

This prime-boost regimen replaced FP9 above with a plasmid DNA encoding the CS protein as a priming agent. In a Phase 1/2a study in the UK, the T cell response was again modest and there was no difference in time to parasitaemia between those vaccinated and the controls after sporozoite challenge [57]. Thus whether DNA/poxvirus or heterologous viral/viral prime-boost approaches are used, the ME-TRAP construct seems to be substantially more immunogenic than the CS construct. This is an important lesson for future projects; one reason for failure of a given prime-boost regimen may be an insufficiently immunogenic antigen and should not necessarily be ascribed to the choice of priming and boosting platforms alone.

#### RTS,S/AS02 + MVA CS

This vaccine regimen was assessed in order to determine if boosting RTS,S/AS02 with MVA encoding the *P. falciparum *CS protein would elicit equivalent antibody titres but greater CMI response than RTS,S/AS02 alone. A Phase 1/2a study in malaria naïve adults in the UK found the prime-boost combination to be safe, but elicited little or no incremental benefit for this regimen compared with RTS,S alone. After sporozoite challenge, vaccine efficacy for complete protection was 2/6 volunteers, the same order of magnitude as protection seen with RTS,S/AS02 alone in previous studies. As with RTS,S/AS02 alone, some volunteers showed a delay in parasitaemia. Disappointingly, MVA-CS did not augment the CS-specific CMI response to RTS,S/AS02 [58]. The objective of this study remains highly relevant as the community searches for alternative viral vectors which could augment the CD8 CMI response to RTS,S.

#### CS DNA immunization

In this strategy, the gene for full length CS protein was inserted into naked plasmid DNA. Multiple Phase 1a studies assessed the safety and immunogenicity of this vaccine, also named VCL-2510, using intramuscular, intradermal and needle-free delivery to induce humoral and cellular responses. While all routes of delivery were safe and elicited some CD8+ T cell responses, no anti-CS antibodies were detected [59-61]. These early studies demonstrated that DNA vaccines alone were substantially less immunogenic in humans than had been observed in small animal models.

#### MuStDO5 (Multi-Stage DNA vaccine Operation, 5 antigens)

This is a combination of DNA plasmids that encode CS, SSP2/TRAP, Exp1, LSA1 and LSA3 adjuvanted with GM-CSF (Granulocyte Macrophage Colony Stimulating Factor). There was no efficacy detected in the challenge study [62] and the investigators have now moved away from DNA plasmid immunization alone.

#### DNA CS/RTS,S/AS02

The DNA vaccine VCL-2510 containing the full length CS gene had been shown to elicit some CD8+ T cell responses and RTS,S/AS02A had elicited CD4+ T cell and anti-CS IgG responses. Vaccination with VCL-2510 followed by boosting with RTS,S/AS02 12-14 months later was safe and immunogenic. The anti-CS antibody and CD4+ response among those boosted was not significantly different from those vaccinated with RTS,S alone, but the DNA/protein prime-boost regimen induced memory CD8+ T cell responses not seen among those vaccinated with only RTS,S [63,64]. However CD8+ T cell γ-interferon responses were not induced as measured by ELISPOT assays conducted on freshly isolated cells.

#### RTS,S/AS02 and TRAP

There is currently no published literature available on this project, but a Phase 2a challenge trial with this vaccine has taken place [1]. It is very important to understand the study design and the outcomes of the project, because combination of CS and TRAP recombinant protein vaccines is a highly logical approach to improving upon RTS,S-mediated efficacy to date. It will be important to determine whether this approach has been adequately tested to failure, or whether further trials are justified with improved TRAP-based recombinant protein constructs.

#### HepB Core-Ag CS VLP

Several clinical studies have assessed this vaccine, also called ICC-1132 or Malarivax, a virus like particle with hepatitis B core antigen genetically engineered to include one B cell epitope and 2 CD4+ T cell epitopes of the CS protein and expressed in *E. coli*. Phase 1 studies of the vaccine adjuvanted in alhydrogel showed acceptable safety with anti-ICC-1132 antibody and IFN-γ induction, but limited malaria specific anti-CS antibodies [65]. Reactivity in functional assays was present in a proportion of vaccinees [66]. When the vaccine was adjuvanted with Montanide ISA 720 and given as a single dose, volunteers seroconverted for IgG to CS but at lower titres than have occurred after 2-3 doses of RTS,S adjuvanted with AS01, 02 or 03 [67]. Although 2 or 3 dose regimens may have been more immunogenic, a single dose ISA 720 regimen was chosen for evaluation in a challenge trial because sterile abscess formation had been seen in non-human primates after 2 doses of ICC-1132 in ISA 720. The challenge trial showed no efficacy against sporozoite challenge [68]. Not long after this study ICC-1132 development was discontinued.

#### CS long synthetic peptide

PfCS102 is a chemically synthesized segment of the *P. falciparum *CS protein containing the C-terminal region with amino acids 282-383. Several Phase 1 studies examined this vaccine construct at varying dosages and combined with different adjuvants. The first Phase 1a study examined PfCS102 adjuvanted with Montanide ISA 720 and alum. Overall, both vaccines were safe and elicited lymphocyte proliferation in all subjects. The ISA 720 adjuvanted vaccine elicited higher antibody levels and enhanced functional activity. Cellular responses were short lived [69]. A second Phase 1a studied examined the vaccine adjuvanted with ISA 720 and GSK's AS02A. In this study AS02A induced stronger humoral and cellular responses [70]. During the 1/2a challenge study, conducted with Montanide ISA 720 alone, all volunteers including controls and vaccinees developed malaria with no difference in time to parasitaemia [71]. There is much debate about the importance of using full length CS, and several groups are now exploring pre-clinical development of full length CS constructs.

#### FP9/MVA & DNA/MVA ME-TRAP

In order to identify the safest and most immunogenic prime-boost regimen to elicit cellular immunity to the liver stage antigen ME-TRAP, multiple non-replicating viral vectors were compared in Phase 1/2a clinical studies in the UK and several field studies in malaria endemic regions [72]. The current clinical programme is focusing on AdCh63 priming and MVA boosting, a regimen confirmed to be highly immunogenic. Prior to this selection, attenuated fowlpox (FP9) or plasmid DNA for priming, followed by MVA boosting had been assessed. Both prime-boost regimens were safe and elicited strong cell-mediated IFN-γ responses, with DNA/MVA ME-TRAP eliciting a stronger response in CD4+ T cells and FP9/MVA ME-TRAP stimulating more CD8+ T cells [73,74], though much weaker CD8 induction than seen with AdCh63/MVA. After a challenge study in malaria naïve adults showed complete protection in some volunteers, Phase 1b studies in Kenyan adults and children assessed the safety and immunogenicity of FP9/MVA ME-TRAP [75,76]. These trials also demonstrated strong IFN-γ responses via *ex vivo *and cultured ELISPOT [77]. A Phase 2 study in Kenyan children resulted in a lower than anticipated T cell response and the vaccine afforded no protection against febrile malaria [78,79]. Earlier trials examining the DNA/MVA ME-TRAP prime-boost vaccination schedule in adults from the UK demonstrated an increase in CD4+ and CD8+ IFN-γ producing cells and a delay in time to parasitaemia compared with homologous vaccination [80]. While field trials in the Gambia of the same vaccine regimen confirmed good T cell immunogenicity, the efficacy in terms of time to first infection was only 10.3% (95%CI -22-34) [81]. These trials also confirmed that priming by naturally acquired infection could be boosted by recombinant poxviruses [82].

#### FP9/MVA polyprotein

This prime-boost strategy combined the non-replicating viral vectors FP9 and MVA. The very long (3,240 amino acid) polyprotein encoded by the vectors consisted of 6 antigens; liver stage antigen-3 (LSA3), sporozoite threonine and asparagine rich protein (STARP), Exp1, Pfs16, TRAP, and LSA1. During the preclinical Phase, the vaccines elicited promising T cell responses to each of the six antigens in certain inbred mouse strains [83], but clinical development ceased due to low efficacy in sporozoite challenge [84]. This was disappointing, because the capacity of poxviruses to encode very large inserts, could be a way to include multiple antigens, if single antigen approaches prove inadequate. It may be that optimization of expression, for example with multiple promoters, could improve upon the results seen with this construct.

#### FMP011/AS01B (LSA-1 E. coli-expressed evaluated with AS02A and AS01B adjuvants)

LSA-1 is a pre-erythrocytic antigen expressed only in infected hepatocytes and LSA-1 antibodies have been associated with reduced risk of malaria blood-stage infection [85]. This candidate used LSA-NRC, an antigen containing T cell epitopes of the N and C terminal regions of LSA-1 and several central amino acid repeats. The antigen was expressed in *E. coli *and used with GSK adjuvants AS01B or AS02A. In a Phase 1/2a trial the vaccines showed acceptable safety among malaria naïve adults, with high titre anti-LSA-1 antibodies. Unsurprisingly for a regimen using a recombinant protein platform, CD8+ T cells were low or undetectable among both groups. Volunteers who received a high dose of either vaccine were challenged, but none were protected and there was no delay in parasitaemia [86].

#### LSA-3

A liver-stage antigen 3 (LSA-3) *L. lactis *expressed recombinant protein has been evaluated in the clinic, adjuvanted with either aluminum hydroxide or Montanide ISA 720 [87]. During a Phase 1/2a study conducted in 2008, one volunteer developed an acute coronary syndrome in temporal association with malaria sporozoite challenge. The coronary syndrome resolved [88] and has led to consensus in the malaria challenge community that formal algorithmic exclusion of those at high coronary risk is appropriate as a precautionary measure in all malaria challenge trials [89]. The safety, immunogenicity and efficacy results of the LSA-3 trial have not yet been published to our knowledge. Proof-of-concept testing for LSA3 remains uncompleted for the time being.

#### *Plasmodium falciparum *blood stage projects

Transfer of immunoglobulin from immune adults to children suffering acute malaria mitigates clinical disease [4,5], but it is not clear whether the targets of naturally acquired protection to severe disease, uncomplicated disease and blood stage infection overlap at a molecular level. Most studies examining incident infection have used parasite detection by microscopy as the endpoint, but it is clear that subpatent infections occur not uncommonly in partially immune people. Thus some studies describing "sterile immunity" may in fact be measuring very effective blood stage immunity that is not sterilizing but maintains parasitaemia at subpatent levels. As discussed earlier, there is little consensus on the degree of contribution of the pre-erythrocytic stages to naturally acquired immunity, but what is clear is that adults with substantial anti-disease immunity become reinfected rapidly on exposure to sporozoites in endemic countries. Furthermore, historical reports of blood-stage challenges of immune adults showed attenuated infections with immunity targeting the blood stages alone [90]. Antigenic polymorphism is a major challenge for most blood stage antigenic targets.

#### MSP-1

Merozoite Surface Protein 1 (MSP-1) is expressed from the onset of schizogony and is involved in erythrocyte invasion by merozoites. Much is known about the structure and function of MSP1 and the possible immune effector mechanisms of MSP1-specific immunity [91]. Anti-MSP-1 antibodies have also been associated with decreased risk of clinical malaria, in an allele-specific manner [92]. The weight of evidence is that IgG induction is more important for MSP1 than cell mediated effector responses.

#### AdCh63/MVA MSP1

This prime-boost strategy utilizes the simian adenovirus 63 vector boosted by MVA, both expressing portions of the *P. falciparum *MSP1 protein, including the dimorphic forms (3D7 and FVO) of the 33kd C-terminal segment as well as MSP1(19) and parts of the N-terminus. Because MSP1 is a very large protein, all clinical projects to date have attempted to identify critical segments of the whole protein for inclusion in the vaccine construct. A Phase 1 trial reported very high mixed CD4/CD8 T cell induction with some IgG induction in addition [93]. A Phase 2a study has also occurred [94,95].

#### FMP010/AS01B

FMP010 is an *E. coli *expressed FVO allele of the 42kD C-terminus of MSP1 and is adjuvanted with the GSK product AS01B. A Phase 1 study examining safety and immunogenicity via growth inhibition assays has occurred [96]. Phase 1b studies were underway in semi-immune populations in Kenya in 2010 [1]. This project follows the previous studies of FMP001/AS02A, a vaccine based on the 3D7 allele of MSP1, and showing good immunogenicity but inadequate clinical efficacy in a Phase 2b trial [97], possibly because multiple vaccine alleles would be necessary to generate a sufficiently broad strain-transcending immune response.

#### MSP-3

Merozoite Surface Protein 3 (MSP-3) is unique in that its anti-parasitic activity was discovered through the exploration of the phenomenon that passive transfer of IgG to naïve volunteers mitigated disease and was associated with reduced disease via antibody dependent cell mediated inhibition (ADCI). Search for the targets of ADCI identified the highly conserved MSP3 antigen and the resulting cytophilic IgG1 and IgG3 anti-MSP3 antibodies as sources of parasitic growth inhibition [98]. A systematic review of immuno-epidemiological studies reported that MSP3 was the blood-stage antigen with the most consistent associations between antibody responses and reduced risk of clinical malaria, in multiple settings [92]. A Phase 1a study in healthy Swiss volunteers demonstrated that the long synthetic peptide MSP3 vaccine elicited a strong humoral response with low reactogenicity when used with aluminum hydroxide adjuvant [99]. Conversely, a Phase 1b trial of semi-immune adults in Burkina Faso resulted in little humoral and modest cell-mediated responses to the vaccine, most likely due to previous exposure and elevated baseline anti-MSP3 antibodies [100,101]. Two Phase 1b trials at sites in Tanzania and Burkina Faso in children aged 12-24 months reported tolerable reactogenicity and good IgG1 and IgG3 cytophilic humoral responses [102,103]. Over 80% of children in both dose groups had a greater than 8-fold increase in IgG subclass titre in the Tanzanian study. Further Phase 1-2 studies in Mali and Burkina Faso have commenced and should be completed during 2011. The reporting of a paediatric Phase 2b study in Mali is awaited [104]. An exploratory analysis of the Phase 1b trial from Burkina Faso of MSP-3 long synthetic peptide adsorbed on aluminium hydroxide reported that rates of clinical malaria in children who received the vaccine in 2 dose groups were lower than in the control group who received hepatitis B vaccine [105]. The sample size was small, and whilst interesting, this finding requires confirmation in a prospective Phase 2 field efficacy study.

#### AMA-1

Apical Membrane Antigen 1 (AMA1) is a blood-stage antigen that aids in orienting the merozoite during invasion of erythrocytes and is also expressed during the sporozoite and hepatic stages. Studies have also shown that anti-AMA1 antibodies tend to be present in those who have acquired natural immunity to malaria [106,107] and repeated natural exposure often leads to high titres of IgG to AMA1. This is in contrast to CS for which even intense exposure induces quite low titres of antibody. The extreme polymorphism of this candidate vaccine antigen suggests that the human immune system exerts a strong selective pressure [108]. These both provides rationale for use of AMA1 as a vaccine in that it is subject to immune pressure, but implies that novel approaches, able to induce strain-transcending responses, will be necessary for useful efficacy.

#### FMP2.1/AS02A

A Phase 1a study with malaria naïve volunteers examining the effect of vaccination with FMP2.1, in which *E. coli *expressed AMA1 (3D7 strain) is adjuvanted with the GSK product AS02A, showed acceptable safety and induction of strong humoral and cell-mediated responses [109]. In a Phase 1b study in Malian adults the vaccine was safe, induced a strong antibody response, but displayed a strongly Th-2 biased cell-mediated immunogenicity [110,111] in contrast to a more balanced Th1/Th2 profile in vaccinated malaria-naïve adults. A Phase 1b dose-escalating study of the same vaccine in Malian children induced at least 100-fold rise in antibody titres compared with baseline anti-AMA1 antibodies, at all three doses [112]. A Phase 2 study in 400 Malian children aged 1-6 reported no significant efficacy against the primary clinical malaria endpoint, but was consistent with allele-specific efficacy against the AMA1 allele contained in the vaccine [113].

#### FMP2.1/AS01B

The FMP2.1/AS01B vaccine candidate uses the same antigen (*E. coli *expressed AMA1 (3D7 strain)) with the AS01B GSK adjuvant. Both adjuvants contain the same amounts of 3-D-MPL (3-deacylated-monophosphoryl lipid A) and QS-21. AS01B is liposome based while AS02A is an oil-in-water emulsion. A Phase 1/2a study in malaria naïve volunteers demonstrated high titres of anti-AMA1 antibody, inhibition of parasite growth *in vitro*, and strong IFN-γ ELISPOT responses. However, after sporozoite challenge, all volunteers became parasitaemic [114]. There was an indication of reduction of the liver-to-blood inoculum for both FMP 2.1/AS02A and FMP2.1/AS01B vaccinees, but the reduction was marginal as assessed by a mathematical model of the qPCR data, with no corresponding difference in pre-patent period as measured by microscopy. There was one notable adverse event 18 days after the second FMP2.1/AS01B vaccination, a rash consistent with a cutaneous immune-mediated hypersensitivity reaction. The rash intermittently recurred at the site of the two previous vaccinations over 3 months [114].

#### AMA-C1/Alhydrogel + CpG 7909

The antigen for this vaccine candidate is *Pichia pastoris *expressed AMA1 with both FVO and 3D7 strains of AMA1. In previous studies with AMA-C1/Alhydrogel, vaccine trials resulted in low titres of anti-AMA1 antibodies and little response in the functional *in vitro *growth inhibitory assay. Thus the vaccine developers evaluated adjuvant combinations of Alhydrogel +/- CpG 7909, a toll-like receptor 9 agonist favouring a Th1 bias and a more potent antibody response. There has been a single case of a serious autoimmune disease known as Wegener's granulomatosis in association with a hepatitis B vaccine adjuvanted with an oligonucleotide similar to CpG 7909. Therefore special attention was paid to possible autoimmune disease in these malaria studies. In a Phase 1a vaccine trial in malaria-naïve adults, addition of CpG was associated with substantially higher IgG responses and increased *in vitro *growth inhibition for both FVO and 3D7 strains [115]. A Phase 1b study in Mali comparing the two vaccines also showed a significantly higher geometric mean antibody titre in the CpG arm as compared to the vaccine without CpG. Difference in the growth inhibition assay activity was not significant, but investigators hypothesize this is due to interference from naturally acquired malaria specific IgG [116]. A Phase 2 study in 300 Malian children aged 2-3 evaluated AMA-C1 with alhydrogel but without CpG. There was no detectable beneficial biological impact of vaccination in terms of reduction in occurrence of parasite density above pre-defined thresholds [117]. Importantly there was an apparent reduction in haemoglobin concentration in AMA1-C1 vaccinees. As this was seen in 2 of 16 reported secondary endpoints, it should be treated with caution. However, this is a biologically plausible outcome of vaccination with an immunogen based on a blood stage antigen and further analysis is warranted. An elegant exploration of allele-specific efficacy in this Phase 2 trial has been published with no indication of an allele-specific effect [118].

#### AdCh63 AMA1/MVA AMA1

This vaccine strategy uses the simian adenovirus AdCh63 expressing AMA1 to prime the immune system and MVA expressing AMA1 to boost. Phase 1a studies for safety and immunogenicity as well as Phase 2a challenge trials have commenced [95,119]. There is now clinical trial data for AdCh63/MVA prime-boost with 3 different antigens. These confirm that this approach is exceptionally immunogenic for CMI, inducing average ELISPOT responses of greater than 1,000 spot-forming cells per million peripheral blood mononuclear cells, with CD8 responses being greater than CD4 responses. It remains to be seen whether the IgG induction will be sufficient for blood stage antigens.

#### EBA175 RII

The EBA175 protein is a blood stage antigen that aids binding of the merozoite to host erythrocytes. The RII region of the protein is highly conserved among the various strains of *P. falciparum*. In a recent Phase 1 study in adult malaria naïve volunteers, a recombinant protein made in *P. pastoris*, adjuvanted with aluminium-phosphate, resulted in a safe and immunogenic response at varying doses [120,121]. Sera from volunteers had anti-EBA175 RII antibodies that demonstrated modest parasitic growth inhibition and inhibition of parasitic binding to erythrocytes *in vitro*. A Phase 1 study in semi-immune adult volunteers in Ghana was underway in 2010 [122].

#### SERA5

This blood stage antigen is expressed during the trophozoite and schizont stages. SE36 is an *E. coli *expressed recombinant protein corresponding to a fragment of the SERA5 antigen. Seroepidemiologic studies in the Solomon Islands found anti-SE36 antibodies to be inversely related to parasite density. In a Phase 1a vaccine trial in malaria naïve Japanese males, the vaccine candidate had acceptable safety and resulted in 100% seroconversion [123]. A Phase 1b trial in semi-immune Ugandan adults has recently been completed [124].

### Combinations of multiple blood stage antigens

#### MSP1 and AMA1 combination: BSAM-2/Alhydrogel + CPG

BSAM-2 is a combination vaccine including MSP1 and AMA1 components. It contains a mixture of recombinant proteins with equal parts *P. pastoris *expressing FVO and 3D7 strains of AMA1 and *E. coli *expressing the FVO and 3D7 strains of MSP1 (42). Each component is adjuvanted with alhydrogel and the solution is mixed with CPG 7909 before vaccination. A Phase 1a study in naïve adults in the US and a Phase 1b study in semi-immune Malian adult is currently taking place [125].

#### MSP1 19 and EBA175 combination: JAIVAC

This combination vaccine consists of MSP1(19) and EBA175, each of which is an *E. coli *expressed recombinant protein adjuvanted with Montanide ISA 720. A Phase 1a clinical trial has recently started in India [126]. This approach targets two antigens with distinct roles in merozoite invasion in the hope that the combination could have additive or synergistic effects over a vaccine based on one target alone [127].

#### MSP-3 and GLURP combination: GMZ2

The GMZ2 candidate vaccine is a *L. lactis *expressed recombinant fusion protein of Glutamate Rich Protein (GLURP) and MSP3, adjuvanted with aluminum hydroxide. A Phase 1a study of the GMZ2 vaccine in malaria naïve German volunteers showed acceptable safety and reactogenicity along with induced anti-GLURP and anti-MSP3 antibodies and memory B-cells [128]. A Phase 1b clinical trial in semi-immune adults from Gabon also showed acceptable safety, a boosted anti-GMZ2 cytophilic IgG response compared with elevated baseline levels, and the induction of memory B-cells [129]. A Phase 1b study in Gabonese children was recently reported [130] with both IgG and memory B cell induction confirmed for GMZ2. Phase 2b studies have been planned for Uganda, Burkina Faso, Ghana and Gabon [1].

### Discontinued/inactive blood stage projects

#### RESA, MSP1, MSP2 (Combination B)

The Combination B vaccine consisted of a mixture of three antigens expressed in *E. coli *and adjuvanted with Montanide ISA 720. The antigens included the N-terminal blocks 1 and 2 of MSP1 (K1 allele), the full length 3D7 allele of MSP2, and a large C terminal fragment of the ring-infected erythrocyte surface antigen (RESA). In a Phase 1/2a study in malaria naïve adults all participants developed parasitaemia and did not experience any delay in growth rate as compared to controls [131,132]. A Phase 1/2b study in children in Papua New Guinea assessed parasite density as an efficacy endpoint. Among children who were not pre-treated with sulphadoxine/pyrimethamine there was a parasite density reduction of 62% (95%CI 13-84), but vaccine efficacy related to prevalence of infection by microscopy, acquisition of new infections and acquisition of clinical malaria was not detected [133-136]. Molecular analyses showed that vaccinees had lower prevalence of parasites carrying a 3D7-type allele and fewer clinical episodes with this variant (corresponding to that in the vaccine). The results provided stimulus for the now discontinued MSP2 bi-allele approach.

#### FMP1/AS02A

Falciparum Malaria Protein-1(FMP1) contains the 42 kDa carboxyl terminus of Merozoite Surface Protein-1 (MSP1) (3D7). The antigen is expressed in *E. coli *with a histidine tag and adjuvanted with AS02A. A Phase 1a trial in malaria naïve volunteers showed the vaccine to have acceptable safety and elicited strong humoral and cellular responses, as well as functional inhibition of parasite growth [137]. Two Phase 1b studies, in Kenya and Mali, showed reasonable induction of anti-MSP1 IgG among semi-immune adults with high titres of IgG at baseline [138,139]. A Phase 1b study in Kenyan children confirmed acceptable safety and immunogenicity with a higher induced titre and lower baseline as expected [140]. However, the Phase 2b trial in 400 Kenyan children aged 12-47 months reported vaccine efficacy of 5.1% (95%CI -26-28) with no reduction in parasite density [97]. The allele-specific efficacy analyses from this trial are awaited as the absence of a strain transcending response would be one logical reason for lack of efficacy.

#### MSP1-C1/AlOH/AlOH + CpG

MSP1-C1 is a combination of FVO and 3D7 strains of MSP1(42) as recombinant proteins expressed in *E. coli*. Original formulations of the vaccine had MSP1-C1 adjuvanted with alhydrogel, but Phase 1 studies found this formulation to be only modestly immunogenic [141] with little activity in a growth-inhibition assay (GIA). In a Phase 1a study in malaria naïve subjects, addition of CpG substantially increased the humoral response to MSP1 [142,143]. We are not aware of planned further development of MSP1-C1 alone, but rather as part of BSAM-2/Alhydrogel + CpG, a MSP1/AMA1 combination [125]. A separate comparison of GIA for equivalent IgG concentrations of anti-AMA1 and anti-MSP1 antibodies confirmed that antibodies to AMA1 are far more potent for growth inhibition *in vitro *[144].

#### MSP2-C1/ISA 720

MSP2-C1/ISA 720 is a combination of 3D7 and FC27 strains of MSP2 as recombinant proteins, adjuvanted with montanide ISA 720. While the vaccine elicited strong humoral and cellular responses in a Phase 1a study, the trial ended prematurely due to increased reactogenicity at the injection site and issues with vaccine stability [1]. The impetus for MSP2-C1 was the results from an earlier trial of Combination B [136], a vaccine which contained one allele of MSP2 and whose efficacy analyses suggested a possible MSP2-related allele-specific effect. It is therefore disappointing that the MSP2-C1 combination was not evaluated in an efficacy study. It would be beneficial to clarify whether this construct has been terminated due to funding priorities or whether it has truly been tested to failure already, funding priorities aside. Increased reactogenicity with ISA 720 is highly likely to be related to the water-in-oil formulation rather than the antigen.

#### AMA1-C1/ISA 720

AMA1-C1 combines the 3D7 and FVO strains of AMA1 recombinant protein, expressed in *P. pastoris*. The combination here was pre-formulated with ISA 720. A Phase 1a study of AMA1-C1/ISA 720 was terminated partway through enrolment due to a documentation problem unrelated to this particular vaccine at the pharmacy where final formulation was conducted. It is difficult to evaluate the incomplete immunogenicity data that was obtained, though the short-lived nature of the induced anti-AMA1 response was notable given the usual depot-like effect of ISA 720. Degradation in droplet size and protein integrity was also noted in long-term stability studies [145]. Both early and delayed local reactions were seen after vaccination, including an injection site nodule in one volunteer.

#### AMA1-FVO (25-545)

In order to compare the reactogenicity and immunogenicity of several adjuvants, this Phase 1a study examined the FVO strain of AMA1 expressed in *P. pastoris *and adjuvanted with either alhydrogel, Montanide ISA 720 or AS02. Generally, all three strategies were acceptably safe, but AMA1 with ISA 720 or AS02 was more reactogenic and displayed higher levels of anti-AMA1 antibodies and IFN-γ and IL-5 cytokines than with alhydrogel [146]. However, due to concerns about whether a single allele of AMA1 was likely to afford relevant clinical efficacy, clinical development of this AMA1 candidate has been discontinued and evolved into the AMA DiCo ("diversity covering") project that has not yet reached clinical trials [1].

#### AMA-1 "Australia"

In this project *E. coli *expressed AMA1 was adjuvanted with Montanide ISA 720. There were low anti-AMA1 antibody responses and only a few volunteers displayed T cell responses. The reactogenicity profile associated with Montanide ISA 720, including local pain and delayed injection site reactions, were also found in this study [147].

#### PfCP2.9 (MSP-1 19/AMA-1 chimera)

PfCP2.9 is a fusion protein containing domain III of AMA1 strain 3D7 and the 19 kDa c-terminal portion of MSP1 strain K1/FVO, expressed in *P. pastoris *and adjuvanted with Montanide ISA 720. Two Phase 1a trials have assessed the safety, reactogenicity and immunogenicity of the vaccine construct at various dosage levels and vaccination schedules. The first 1a trial, examining high doses of the vaccine, demonstrated high anti-PfCP2.9 antibody titres but displayed some local reactogenicity [148]. The second Phase 1a trial, assessed the vaccine at lower doses and with wider gaps in the vaccination schedule. The antibody titres remained high with reduced reactogenicity, but GIA and IFA activity were suboptimal and the immunogenicity was markedly lower than seen in non-human primates [149].

#### GLURP (85-213)

Anti-GLURP antibodies have been associated with a reduced risk of clinical disease in those naturally exposed, and as with MSP3, cytophilic IgG subclasses are implicated in possible protection. A Phase 1a trial utilizing a GLURP long synthetic peptide adjuvanted with either aluminium hydroxide or Montanide ISA 720 showed both a strong cell-mediated and humoral response, especially for anti-GLURP IgG1. However, both formulations of the vaccine displayed a dose-dependent increase in adverse events at the injection site and 7/18 volunteers in the ISA 720 group did not receive a third immunization due to predefined withdrawal criteria [150]. Evaluation of GLURP continues as part of the GMZ2 construct.

### Combination vaccines including multiple life-cycle stages

#### NMRC-M3V-D/Ad-PfCA Prime/Boost & NMRC-M3V-Ad-PfCA

These approaches are DNA-based vaccination combining the CS pre-erythrocytic antigen with the AMA1 blood-stage antigen (now known also to also be expressed in other stages of the life-cycle). The initial strategy was to use DNA encoding CS and AMA1 proteins to prime, then boost with adenovirus 5 vectors expressing the same antigens. Publication of the results of the Phase 2a challenge study is awaited [151]. A Phase 1 trial was conducted in US with adenovirus 5 vectors encoding CS and AMA1. Very high frequencies of CD8+ T cells were induced [152], but no sterile protection was afforded in a follow-on Phase 2a trial using the CS component alone [153]. These approaches confirm the promise of Ad5 for induction of CD8 responses in humans. Some refinements to this approach are in pre-clinical development [1].

#### CS, AMA1 virosomes (PEV301,302)

Synthetic peptides were manufactured representing fragments of malaria proteins thought to be good immune targets. These are displayed as phospatidylethanolamine (PE)-peptide conjugates on the surface of IRIVs (immunopotentiating reconstituted influenza virosomes). PEV301 is the virosome with the synthetic CS protein conjugate UK39, and PEV302 virosome has the synthetic AMA1 protein conjugate AMA149-C1. IRIVs have already been used for licensed influenza and hepatitis A vaccines and, in various guises, can deliver multiple antigens to focus on CD4, CD8 T cell or antibody responses. A Phase 1a study in healthy malaria naïve adults demonstrated that volunteers immunized with a mix of both PEV301 and PEV302 developed anti-AMA149-C1 and anti-UK39 antibody titres [154]. Further immunogenicity studies on the PEV301 portion of the vaccine revealed induced *in vitro *inhibition of sporozoite migration and hepatocyte invasion [155]. In a Phase 2a experimental sporozoite challenge trial in malaria non-immune Caucasian volunteers, vaccine-related partial but modest protection against sporozoite challenge was observed in terms of a delay in time to parasitaemia [156], although no sterile protection was observed. A recently completed Phase 1b vaccine trial in semi-immune Tanzanian adults and children confirmed the safety and immunogenicity of the platform. In addition, an exploratory analysis showed a reduced incidence of clinical episodes of malaria. Whilst interesting this requires confirmation in field efficacy studies [157]. There has been a change of ownership of the biotech company with potential implications for the future application of this promising technology to malaria.

### Discontinued combination vaccines including multiple life-cycle stages

#### Spf66

Spf66 is a vaccine candidate developed in Colombia in the late 1980s. It is a synthetic peptide consisting of CS and merozoite surface protein 1 (MSP1) epitopes adjuvanted with alum, and more recently tested with QS-21. Several studies in multiple Phases and countries have assessed this vaccine and while overall the vaccine was felt to be safe, the immunogenicity and vaccine efficacy have been relatively low [158]. Phase 3 studies in South America reported a vaccine efficacy of 28% (95%CI 18-37) for clinical malaria [159], but the African and Asian studies taken together failed to show useful efficacy for this vaccine [160-169]. There was much debate about the interpretation of the South American results because of the many differences in trial design between the first studies and those in Africa and Asia. This is an important example of the fact that malaria field efficacy data should be obtained using standardized consensus case definitions, endpoints and trial designs in a variety of epidemiological settings among various age groups, and then be applied only to the settings and age groups in which it was obtained, because of the major heterogeneities of malaria epidemiology, immunity and vaccine efficacy. When vaccine efficacy varies from trial to trial, manufacturing consistency between vaccine lots could be a contributory factor.

#### RTS,S/AS02 (CS) and FMP-1/AS02 (MSP1)

Combination of RTS,S with other promising approaches has long been one stated aim of the RTS,S development programme. In this Phase 1/2a trial, researchers concomitantly administered FMP-1/AS02 and RTS,S/AS02A. Efficacy of RTS,S/AS02 was similar in co-administration with FMP1/AS02 compared to RTS,S alone. This indicated that there was no detectable immunological interference between the two components, and that FMP1 gave no protection in the challenge model as detected by microscopy [170].

### Sexual stage vaccines

Currently there are a few sexual stage vaccines in preclinical development and at least one Phase 1 trial is underway. As the vaccines are intended to block the life-cycle in *Anopheline *mosquitoes, vaccine efficacy needs to be measured as a reduction in community level transmission of malaria, through cluster randomized trials. Due to the logistic challenges with conducting large cluster randomized trials, the availability of data for decision-making earlier in the development pathway could greatly facilitate progress with these vaccines. Intermediate proof-of-principle efficacy trials may be possible with the emphasis on demonstrating a reduction in infectivity of humans for mosquitoes. A valuable membrane-feeding assay (MFA) enables investigators to assess the ability of sera raised against sexual stage antigens to reduce infection of mosquitoes on exposure to gametocyte-infected blood, but the link between immune responses in individuals and effect on transmission is not known. Efforts to qualify, and if possible to validate, this assay will be important for vaccine development.

Given the disappointing results of many blood stage vaccine projects, and the emphasis on malaria eradication as a long-term aim by some funders, sexual and mosquito stage vaccine R&D funding may well increase over the next 5-10 years.

Previous clinical trials of sexual stage vaccines that have been discontinued involve ookinete antigens Pfs25 from *P. falciparum *and Pvs25 from *P. vivax*. Phase 1 studies examined the antibody response to the vaccine as well as activity in MFA. *Saccharomyces cerevisiae *expressed Pvs25, adjuvanted with alhydrogel, elicited low titre anti-Pvs25 antibodies and little transmission blocking activity [171]. A second Phase 1 study examining Pvs25 and Pfs25 adjuvanted with Montanide ISA 51 induced some antibody response and transmission blocking activity. The study was stopped due to unacceptable reactogenicity most likely related to the adjuvant [172]. In both studies and in pre-clinical work by the same group there is a consistent correlation between titre of anti-Pfs25 antibody and MFA activity [173].

Two of the sexual stage vaccine projects in preclinical development are based on the Pfs25 antigen. Preclinical studies in mice have shown that when Pfs25 is chemically conjugated to recombinant *Pseudomonas aeruginosa *ExoProtein A (EPA) and adjuvanted with alhydrogel, both the anti-Pfs25 antibody response and transmission blocking activity are significantly higher than with Pfs25/alhydrogel alone [174,175]. A Phase 1 clinical trial of this vaccine candidate began in the latter part of 2011 [176]. Given that sexual stage and mosquito antigen vaccines will need to be administered across a wide age range commensurate with the human infectious reservoir in each setting, robust safety is a key feature of the desired target product profile. It is therefore highly encouraging that the pre-clinical studies with Pfs25-EPA indicate that alhydrogel may be sufficient as an adjuvant. Avoiding the many challenges associated with novel adjuvants, where possible, may be advisable for this group of vaccines.

Preclinical studies examining multimeric Pfs25 self-conjugates also showed an increase in anti-Pfs25 antibody levels and reduction of oocyst numbers in the mosquito midgut [13].

### *Plasmodium vivax *vaccine projects

Control of mosquito vectors that support *P. vivax *has proven more challenging than *P. falciparum *in many settings, and *P. vivax *transmission may persist after *P. falciparum *has been eliminated because of its capacity to cause relapse from hypnozoites in the liver. Field trial design is complicated by the fact that *P. vivax *vaccine evaluation will most likely occur in co-endemic settings and the fact that distinguishing hypnozoite reactivation from new infections is not straightforward. Additionally the sporozoite challenge model is less well established for *P. vivax*, partly because culture of *P. vivax *gametocytes is problematic.

As with sexual stage and mosquito antigen projects, *P. vivax *is likely to receive additional R&D funding in the next 5-10 year cycle, particularly if *P. falciparum *transmission, morbidity and mortality continue to fall. Thus although there is a little clinical activity to report now, this is likely to change [177].

#### VMP001/AS01B

VMP001/AS01B is currently the only vaccine in clinical evaluation that is designed to protect against *P. vivax*. In contrast to *P. falciparum*, the immunodominant B cell epitopes exhibit dimorphism in *P. vivax*. VMP001 includes the repeat region from the two alleles (VK210 and VK247) as well as N and C terminal fragments. It is an *E. coli *expressed recombinant protein adjuvanted with AS01. A Phase 1/2a challenge trial occurred in US volunteers at the end of 2010 [14].

## Discussion and Conclusion

Over two decades on from the first clinical trials of vaccines for malaria, much has been learned about the pipeline from discovery research in the laboratory to successful conduct and analysis of large-scale field studies (see table 1), and one product is undergoing Phase 3 studies with a view to licensure. In this period, substantial progress has been made in evaluating many antigens and scientists have learned much about the need to meet requirements of developers in establishing clear product profiles.

**Table 1:** Lessons learned in terms of safety, immunogenicity, efficacy and trial methodology from malaria vaccine research over the last 5-10 years

Safety	Reactogenicity is higher with water-in-oil emulsions (e.g. ISA 720) when compared to marketed adjuvants (alhydrogel) or a marketed virosomal platform.
	
	Safety and reactogenicity in semi-immune populations living in endemic areas has not been higher than in naïve populations, and is often lower.
	
	Safety and reactogenicity in young children has not been worse than in adult populations
Immunogenicity	DNA alone is poorly immunogenic
	
	Oil in water emulsions (AS01, AS02) and water in oil emulsions (ISA 720, ISA51) are more immunogenic than alhydrogel for recombinant monomeric protein
	
	In general, there is little induction of CD8 cells in humans (with the exception of certain adenovirus containing regimens)
	
	In general, there has been little clinically significant interference between the malarial antigen and EPI vaccine antigens

Efficacy	Only RTS,S-based vaccines have repeatedly shown efficacy to reduce morbidity in endemic areas

	Highly polymorphic blood-stage antigens have tended to lead to allele-specific efficacy, but poor efficacy against the population of circulating strains

	Multiple episodes of malaria takes priority over time to first episode for public health assessment in clinical malaria vaccine trials.

Methodology	Demonstration of effect in *in vitro *studies (growth-inhibition assay in particular) or animal studies have not been shown to correlate well with efficacy results in the field

	Human challenge studies (Phase IIa trials) have been validated for the screening of pre-erythrocytic vaccines by the RTS,S results

	It is agreed that every Phase IIb/III vaccine trial design includes a commercialized vaccine that will benefit the control group as comparator and that any trial subject receives at least the standard package of preventive measures (LLIN and others) implemented in the country.

	Methodological and ethical issues would arise in testing of new malaria vaccines in the field if a licensed malaria vaccine had become a standard preventive measure in a given setting.

	Methodological and feasibility issues are arising to test new vaccines in the field because malaria morbidity is considerably decreasing in areas where trial sites are in place.

	There is as yet no formal trial design to assess the efficacy of sexual stage and mosquito antigen vaccines prior to large scale cluster randomized trials; specific baseline epidemiological studies are required for sample size calculations and trial design for possible Phase IIb trials in this area

Compared with other infectious diseases of major global importance such as HIV and tuberculosis, malaria vaccine research is facilitated by the availability of a clinical challenge model and a high attack rate in endemic areas, enabling definitive assessment by human experimentation for vaccines that prevent infection. There is consensus for testing the efficacy of pre-erythrocytic vaccines in sporozoite challenge studies, and an emerging consensus that some evidence of effect as evidenced by a degree of sterile protection or at least a reduced parasite growth rate is required as a pre-condition for moving asexual stage vaccines to expensive large-scale clinical trials, as other measures such as growth-inhibitory assays have yet to fulfill their promise as surrogate markers of protection. WHO is facilitating a harmonization process for sporozoite challenge trials with consensus Standard Operating Procedures and documents available to guide design and conduct of such trials.

There is now consensus on many of the case definitions, endpoints and best analytical methods for measurement of reduction of morbidity conferred by pre-erythrocytic vaccines [178]. WHO has requested that data on multiple episodes of malaria, and time at risk, is presented divided into time periods to enable evaluation of long-term follow-up data [179], as impact on multiple episodes of malaria is the most important clinical/out-patient malaria endpoint from the public health and policy perspective. Methodological areas which remain the subject of ongoing work include: statistical methods for examining the duration of protection of vaccines taking into account the major but unmeasured heterogeneities of risk; case definitions and endpoints for blood-stage vaccine efficacy trials; trial designs for evaluation of reduction in infectivity of humans for mosquitoes.

Profiles for products targeted to reduce the global burden of malaria have focused on the desirability of reducing mortality and morbidity in vulnerable groups of young children and pregnant women [180] living in endemic areas, whereas targets for military and traveler populations focus on complete prevention of infection, even for relatively short periods after vaccination. It is highly likely that different immune mechanisms operate to prevent infection or modify disease but in neither case is the critical protective immune response for these target groups known or understood. Promising effects of RTS,S have encouraged investigators and funding agencies to raise the longer term target product threshold efficacy to the order of 80% for second generation products, yet still with a longer term "gold standard" aim for complete protection from clinical disease.

More recently, thanks to some successful malaria reduction campaigns in parts of Africa and the critical advocacy of Bill and Melinda Gates, the malaria community is considering the research and other requirements for a strategy leading to eventual eradication of malaria [11]. Of course this has always been seen as the ultimate goal but priority in the past four decades has been given to reducing the disease burden in regions with highest endemicity. The re-emergence of debate on eradication has not reduced the priority that scientists give to vaccines for potential impact, but has caused vaccines to be seen through another lens for their potential to reduce disease transmission. Vaccines directed against any stages of the parasite could have impact on transmission, with sexual stage vaccines targeting only this activity, and vaccines against mosquito gut having similar potential.

There was some concern two decades ago that vaccines designed to prevent immunity in travelers or the military may be of limited benefit to children of endemic areas since immunity against malaria is known to be species and stage specific. It is believed that a pre-erythrocytic vaccine designed to prevent infection in a non-immune individual would need to be highly effective for impact (well over 80% sterile protection) since a single parasite reaching the blood stream could lead to lethal infection. This however may not be true for semi-immune individuals when an effect of reducing the burden of parasites reaching the bloodstream may be of benefit in reducing uncomplicated malaria and severe morbidity. The better than expected effects of RTS,S vaccine in children of endemic areas support this hypothesis, and suggest that this vaccine could be achieving in some of the population the desired goal of converting the immune status of infants of endemic areas from that of a newborn to that of a three year old child who is protected, albeit incompletely, from clinical disease despite recurrent infection (concomitant immunity).

Early success with the pre-erythrocytic vaccine RTS,S and the call for malaria eradication have led some funders to reduce priority and funding for vaccines directed against blood stages. Most in the field argue that the considerable evidence for protection directed against blood stages, such as passive transfer experiments, validate the approach of seeking to combine the best candidates from more than one stage. A major consideration is to determine criteria for advancing particular candidates.

Various factors have been used to decide on moving candidates to clinical trials but as none has proved reliable, better ways of making this assessment and down-selection are needed. Animal models are not optimal and immune responses in rodents or non-human primates have not so far proved to be reliable indicators. Highly variable antigens with multiple alleles are obviously targets of the immune response under natural challenge, and vaccine studies of AMA-1 and MSP 2 suggest that allele-specific effects can be achieved. However, the task of making a construct that would induce protection against a multitude of variants could be insurmountable. On the other hand, invariant antigens that are critical for invasion could be good targets if they could be made immunogenic. There is growing consensus that in order to progress an antigen to field trial stage, there is a requirement for the candidate to have demonstrable impact on parasite growth in non-immune individuals. Recent advances in antigen delivery systems using viral like particles, novel adjuvants, and alternate delivery systems, such as recombinant viral delivery and heterologous prime boosting have demonstrated capacity to induce effective responses that are not seen following natural infection and treatment or recovery.

Advances in technology have now provided proof of principle (in other systems as well as malaria) that the combination of priming with DNA and boosting by viral delivery, or priming and boosting with two different viral delivery systems can induce protection in a proportion of individuals. Studies of protected and non-protected individuals may enhance understanding of the critical immune response and give a guide to correlates of protection that may then potentially be inducible with simpler regimens. Malaria vaccine researchers follow developments in HIV and tuberculosis with great interest, considering the similar challenges of understanding the non-sterilising clinical immunity that occurs at stages of these three diseases. All share the goal of understanding mechanisms for inducing long-lived immunological memory and how new and improved technologies could achieve this goal

With regard to adjuvant selection, the ICC-1132 project described in this review [65-68] serves as a useful case study of some general points. First, alum-adjuvantation has been repeatedly demonstrated to be inadequate for inducing the desired response to monomeric recombinant malaria proteins. However if alum-adjuvantation were to be sufficient in specific cases this would probably represent the best adjuvant approach in many ways, and there is some hope that with increased immunogenicity of protein conjugation or particulate technologies, alum-adjuvantation might suffice in some cases. Secondly, promising immunogenicity in mice, as was seen with ICC-1132, has often not translated to the clinic. Thirdly the strongest clinical experience with adjuvants is with GSK's proprietary adjuvants such as AS02 and AS01. Given the issues with access to these proprietary adjuvants, groups have often opted for use of water-in-oil adjuvants such as ISA 720 or ISA 51 and these tend to give good results in pre-clinical experiments. However, an unfavourable combination of issues with chemical modification of antigens that can compromise stability, a lack of consistency for bedside formulation and at times unacceptable local reactogenicity have tended to terminate development of many water-in-oil formulations in the malaria field. There is sufficient experience with these water-in-oil emulsions now to recommend alternate formulations. Thus access to sufficiently potent adjuvants suitable for clinical use and without proprietary limitations remains a key rate-limiting factor in malaria vaccine development.

Many lessons have been learned about the long process of establishing field trial sites, gaining regulatory approval, establishing Ethics Committees, and completing analysis. Incidence of malaria invariably falls with the intensive surveillance required for clinical analysis in well-monitored field sites and trial sample sizes are necessarily increased considering the decreased incidence of malaria for test and control groups, when gold standard control measures are applied for all participants. In addition, malaria transmission has fallen in the region of several field trial sites, probably due to the success of national malaria control programmes. Investigators have recognized the need to be prepared to increase the duration or geographical scope to achieve the required numbers of enrolments. It is essential to develop local regulatory and ethics capacity for local trials. Communication is critical for informing participants and communities about the goals of studies that in many cases would be better described as "experimental medicine" or "trials in humans" rather than "vaccine trials", in order not to raise artificially high expectations of outcomes. Establishing these basic supports is very important for the large number of future trials to examine various combinations and the duration of efficacy. So far there has been no evidence of disease enhancement, but researchers must continue to be vigilant, and also be aware of potential rebound morbidity following temporary interruption of clinical infections.

Considering the devastating impact of the disease, global resource allocation has been modest, particularly now that the RTS,S success confirms that malaria vaccination can reduce paediatric morbidity in the field. In the years ahead it will be appropriate to aim for a vaccine with 80% or higher efficacy and substantial impact on transmission as envisaged by the malaria vaccine technology roadmap, as a 2025 goal, and malaria eradication R&D agenda initiatives.

## Competing interests

All authors declare that they have no competing financial interest. GB, BG and VM have previously acted as investigators on the following malaria vaccine trials: GB, Combination B trials in Papua New Guinea; BG, Phase Ib in Papua New Guinea adults. Phase IIb in children of Combination B, Phase Ia and IIa of PfCS102 in adults in Switzerland, Phase Ia in Swiss adults and Ib in Tanzanian children and adults of the Pevion virosomes; VM, Phase Ia, IIa, Ib, IIb of DNA/MVA and FP9/MVA ME-TRAP in Oxford and The Gambia.

## Authors' contributions

LS carried out the rainbow table update upon which this manuscript is based. LS obtained and read all key references, and clinical trial registry information. LS wrote the first draft of the manuscript. VM coordinated writing of the second and final drafts of the manuscript and revised the manuscript in response to reviewer's comments. GB and VM wrote the discussion and edited the second and final draft. BG edited and commented on the second and final drafts and compiled the table.
